# Synaptopathy Mechanisms in ALS Caused by C9orf72 Repeat Expansion

**DOI:** 10.3389/fncel.2021.660693

**Published:** 2021-06-01

**Authors:** Agnes L. Nishimura, Natalia Arias

**Affiliations:** ^1^Department of Basic and Clinical Neuroscience, UK Dementia Research Institute, Maurice Wohl Clinical Neuroscience Institute, Institute of Psychiatry, Psychology and Neuroscience, King’s College London, London, United Kingdom; ^2^INEUROPA, Instituto de Neurociencias del Principado de Asturias, Oviedo, Spain

**Keywords:** synaptic transmission, dendrites, dendritic spines, synaptopathies, neurodegeneration, amyotrophic lateral sclerosis

## Abstract

Amyotrophic Lateral Sclerosis (ALS) is a complex neurodegenerative disease caused by degeneration of motor neurons (MNs). ALS pathogenic features include accumulation of misfolded proteins, glutamate excitotoxicity, mitochondrial dysfunction at distal axon terminals, and neuronal cytoskeleton changes. Synergies between loss of C9orf72 functions and gain of function by toxic effects of repeat expansions also contribute to C9orf72-mediated pathogenesis. However, the impact of haploinsufficiency of C9orf72 on neurons and in synaptic functions requires further examination. As the motor neurons degenerate, the disease symptoms will lead to neurotransmission deficiencies in the brain, spinal cord, and neuromuscular junction. Altered neuronal excitability, synaptic morphological changes, and C9orf72 protein and DPR localization at the synapses, suggest a potential involvement of C9orf72 at synapses. In this review article, we provide a conceptual framework for assessing the putative involvement of C9orf72 as a synaptopathy, and we explore the underlying and common disease mechanisms with other neurodegenerative diseases. Finally, we reflect on the major challenges of understanding C9orf72-ALS as a synaptopathy focusing on integrating mitochondrial and neuronal cytoskeleton degeneration as biomarkers and potential targets to treat ALS neurodegeneration.

## Understanding Amyotrophic Lateral Sclerosis as A Synaptopathy: The Role of C9orf72

### ALS as a Synaptopathy

Amyotrophic lateral sclerosis (ALS; also known as motor neuron disease) has a remarkably variable clinical presentation and progression, with ~75% of patients presenting upper and lower limb weakness (Vucic et al., 2014). The remaining 25% of patients present bulbar signs and symptoms, including impairment of speech and swallowing difficulties (Vucic et al., 2014). Approximately 20–50% of ALS patients also present frontotemporal dementia (FTD) symptoms, which obscure diagnosis and prognosis (Vucic et al., 2014; Genc et al., 2017). Despite variable clinical presentations, cortical post-mortem pathological features are highly conserved across sporadic and familial forms of ALS (Genc et al., 2017). These include dendritic and synaptic degeneration in the cortex and corticospinal motor neurons (CSMNs; Fogarty, 2019).

**Figure 1:**
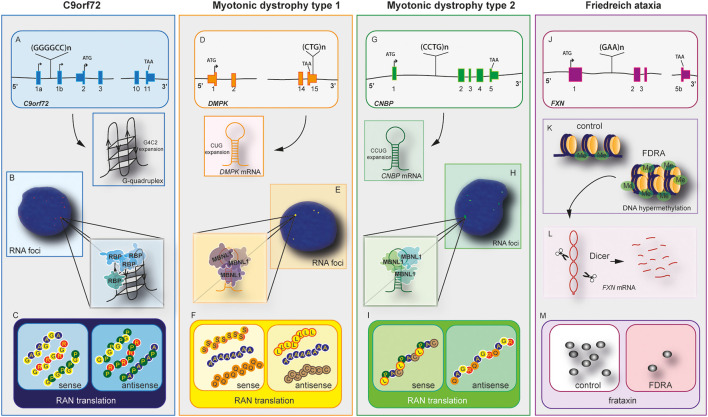
C9orf72 and other repeat expansion disease mechanisms. Nucleotide repeat expansions located at intron or untranslated regions (UTRs) cause three main disease-related features, haploinsufficiency in C9orf72 **(A)**, myotonic Dystrophy type 1 (DM1; **D**), myotonic dystrophy type 2 **(G)**, and Friedreich’s ataxia (FRDA) **(J)**. mRNAs containing repeat expansions form G-quadruplex (C9orf72) and RNA hairpin structures (myotonic dystrophy type 1 and 2). These RNA hairpins will accumulate and form RNA foci **(B,E,H)**, which will sequester RNA binding proteins (RBPs) including hnRNP-H in C9orf72 and Muscleblind like Splicing Regulator 1 (MBNL1) in myotonic dystrophy type 1 and 2. Repeat-associated non-AUG (RAN) translation produces peptides from the sense and antisense strands in C9orf72 **(C)**, myotonic dystrophy type 1 **(F)**, and myotonic dystrophy type 2 **(I)**. The exception lies in FRDA disease mechanisms. In this disease, the repeat expansion causes DNA hypermethylation and gene expression dysregulation of neighboring genes **(K)**. The mRNA forms hairpins, which are degraded by Dicer **(L)**, leading to downregulation of frataxin **(M)**. In this disease, RNA foci and RAN translation were not observed.

Cortical synaptic degeneration landmark has inspired Braak and colleagues to classify ALS as a disease of large axons neurons with discrete stages of trans-synaptic spread (Braak et al., 2013). This classification suggests that the disease starts in CSMNs, descends to MNs, and progresses to extramotor areas (Braak et al., 2013; Brettschneider et al., 2013). This pathological insight is consistent with cortical hyperexcitability as an early clinical feature of ALS (Vucic et al., 2014). Moreover, the trans-synaptic spread hypothesis suggests a mechanism of spreading misfolded protein aggregates to distant populations of neurons through a prion-like transmission mechanism (Braak et al., 2013). This is consistent with the hypothesis of ALS being a synaptopathy (Fogarty, 2019). Synaptopathy is a broad definition of diseases with synaptic dysfunction, regardless of the disease mechanisms (Lepeta et al., 2016). In the broadest definition, synaptopathy encompasses a wide range of features, which in due course, will lead to synaptic dysfunction. These features include changes in Ca^2+^ levels at synapses, glutamate excitotoxicity, structural changes in pre- and postsynaptic anchoring proteins, altered synaptic structure and function, which is often associated with dendritic spine loss, dysfunctional neurotransmitter release (quantal content and frequency), impaired maintenance, and regeneration of axons by Schwann cells, and cognitive deficit (Lepeta et al., 2016; Fogarty, 2019). Furthermore, synaptopathies lead to neuronal loss, mitochondrial dysfunction, accumulation of misfolded proteins associated with defective proteostasis, and defective neuromuscular junctions (NMJ; Figure 1; Wishart et al., 2006; Lepeta et al., 2016; Fogarty, 2019).

In this line, several studies have demonstrated abnormal morphology and function of synapses in ALS, with the nature of changes dependent on the disease timeline (Genc et al., 2017; Fogarty, 2018). Moreover, notable changes observed in ALS post-mortem samples are neuronal loss, change in the dendritic spine density, and morphology in excitatory neurotransmission sites, particularly in pyramidal cells such as CSMNs (Genc et al., 2017; Fogarty, 2018). This feature is also observed in different rodent models of ALS (Jara et al., 2012; Fogarty et al., 2017). In this review, we will discuss how changes in different pathways will lead to synaptic dysfunction in C9orf72-ALS pathology.

### C9orf72 Role in Synaptic Dysfunction

The identification of an expansion of the hexanucleotide repeat GGGGCC (or G4C2) in intron 1 of the *C9orf72* gene has opened a new field of investigation on long non-coding RNAs and neurodegenerative diseases. The G4C2 expansion was first identified in 2011 in patients with ALS and FTD (DeJesus-Hernandez et al., 2011; Renton et al., 2011). Despite *C9orf72* being initially involved in these two diseases, G4C2 expansions have also been observed in other neurodegenerative conditions including Creutzfeldt–Jakob disease, progressive supranuclear palsy, ataxia, Huntington disease-like syndrome, corticobasal syndrome, Parkinson’s disease, and Alzheimer’s disease (Woollacott and Mead, 2014).

Three plausible pathogenic mechanisms have been linked to the G4C2 expansion and they include: (a) loss of function mechanism due to G4C2 repeat expansion leading to downregulation of C9orf72 protein expression; (b) toxic gain of function by recruitment of other RNA-binding proteins into G4C2 RNA foci; and (c) aberrant dipeptide repeat proteins (DPR)s produced by repeat-associated non-AUG (RAN) translation. Six DPRs are produced from both strands generating poly-glycine-alanine [poly (GA)], poly-glycine-arginine [poly (GR)], poly-glycine-proline [poly (GP)] from the sense strand and poly-proline arginine [poly (PR)], poly-proline-alanine [poly (PA)], and poly-proline-glycine [poly (PG) or poly (GP)] from the antisense strand. These DPRs display different toxic properties in distinct animal and cellular models (Ash et al., 2013; Mori et al., 2013; Zu et al., 2013; Mizielinska et al., 2014; Haeusler et al., 2016; Lee et al., 2020) and their roles in synaptic dysfunction will be further discussed.

Little was known about the role of C9orf72 protein when it was first associated with ALS and FTD. Levine et al. (2013) reported that C9orf72 protein sequence and structure analyses share distant homology to Differentially Expressed in Normal and Neoplasia (DENN; Levine et al., 2013). The DENN protein is a GDP/GTP exchange factor (GEF) that activates Rab-GTPases. Thus, the homology to DENN protein suggests that C9orf72 may regulate membrane traffic and promote Rab-GTPase switches. Rab-GTPases are distributed in the soma and neurites and specific subtypes of Rab-GTPases, such as RAB3 and RAB8, are enriched at synapses in flies (Kiral et al., 2018). Atkinson et al. (2015) reported that C9orf72 protein is localized in different compartments in the cells during mouse development (Atkinson et al., 2015). At the embryonic stage and P1, C9orf72 forms small puncta throughout the neuropil, whereas at P7 and P56, C9orf72 is diffused in the nucleus and soma. Furthermore, C9orf72 protein is found in the synaptosome fraction, and the C9orf72 isoform 1 is enriched in the synaptic fraction in the adult mouse brain. Corroborating these data, Frick et al. (2018) showed similar findings using novel C9orf72 antibodies in wild-type mouse brain tissues (Frick et al., 2018). These authors observed that C9orf72 is mainly located in pre-synaptic compartments in the mouse brain and interacts with the RAB3 protein family (Frick et al., 2018). C9orf72 forms complex with SMCR8 and WDR41 (Sellier et al., 2016; Sullivan et al., 2016; Yang et al., 2016) with a function as GDP/GTP exchange factor for RAB3, RAB8a, RAB39b, and ULK1, suggesting a potential involvement in synaptic transmission and autophagy (Sellier et al., 2016; Yang et al., 2016; Frick et al., 2018; Xiao et al., 2019). Furthermore, synaptic fractionation of C9orf72 knockout mouse brain revealed that C9orf72, SMCR8, and RAB39b proteins were significantly depleted, with concomitant upregulation of GluR1 (Xiao et al., 2019). In this line, it has been shown that downregulation of C9orf72 partly impairs autophagy leading to accumulation of TDP-43 and p62, evidence of misfolded proteins accumulation (Sellier et al., 2016). Moreover, this role of C9orf72 protein in synaptic dysfunction has been supported by the localization of this protein in pre- and post-synaptic compartments (Xiao et al., 2019).

Studies using induced pluripotent stem cells (iPSCs) derived from patients with the *C9orf72* expansion revealed that mutant lines displayed altered synaptic activity. Sareen et al. (2013) reported that neurons derived from C9orf72 patients were less excitable than control neurons, whereas Devlin et al. (2015) demonstrated that mutant C9orf72 lines presented hyperexcitability followed by a progressive loss of synaptic activity. Furthermore, two independent reports showed C9orf72 fly models presented dysfunctional neuromuscular junction transmission, with fewer active zones (Freibaum et al., 2015; Zhang et al., 2015). Herranz-Martin et al. (2017) described mice lines overexpressing 102×G4C2 repeats mediated by adeno-associated virus (AAV) and observed abnormalities in the NMJ. Lower acetylcholine receptors were observed in the C9orf72 mice compared to controls.

Interestingly, dipeptide repeats may also play a role in synaptic dysfunction. Xu and Xu (2018) induced the expression of different DPRs in *Drosophila* models. They observed that poly (GR) and poly (PR) overexpressing flies presented altered synaptic buttons at NMJs and degeneration of glutamatergic neurons due to excitotoxicity mechanisms. In contrast, Jensen et al. (2020) observed that poly(GA) peptides are located in neurites and are less mobile in overexpressing poly(GA) mouse models.

Finally, it is important to point out that despite C9orf72-ALS presenting features compatible with synaptopathy (glutamate excitotoxicity, accumulation of misfolded proteins, and mitochondrial dysfunction at distal axons) a few disease features deviate from the synaptopathy hypothesis. Such an example is the contribution of neuroinflammation in the ALS disease process (Fogarty, 2019). This inflammatory reaction is a result of a complex cascade of events characterized by infiltration of T-lymphocytes and macrophages and activation of microglia and reactive astrocytes (Philips and Robberecht, 2011). It is believed that neuroinflammation is activated to combat the mutant misfolded protein, however, in a bizarre turn of events, it becomes a hazardous process that contributes to neuronal damage instead (Philips and Robberecht, 2011). Furthermore, the limitations of these collective studies lie on their descriptive nature, represented by a snapshot of temporal and spatial changes. This is partially due to the experimental challenges and limitations in using cellular models to investigate synaptic dysfunction *in vitro*. The combination of electrophysiology and neuroimaging studies in patients and in animal models may provide more insights into synaptic dysfunction in C9orf72-ALS. Whether synaptic dysfunction is causal or a consequence of disease progression, more research into this field is needed to understand how synaptic dysfunction contributes to C9orf72 pathology in ALS.

## The Role of Synaptic Neuroplasticity and Mitochondria Dysfunction in C9orf72 Neurodegeneration

Neuroplasticity or neuronal plasticity involves structural and functional adaptations of neuronal circuits to changes due to learning and memory, environmental influences, and brain damage (Malenka and Nicoll, 1999; Cheng et al., 2010). Structural plasticity processes in the mature nervous system are known as synaptic plasticity and include dendritic spine growth and synaptogenesis (Mattson, 2007). Long–term potentiation (LTP) and long–term depression (LTD) are relevant examples of neuroplasticity (Malenka and Bear, 2004). During LTP and synaptic activation, several studies have reported changes in mitochondria (Mattson and Liu, 2003), including energy production, calcium buffering and pump activity, and mitochondrial gene expression (Wieraszko, 1982; Stanton and Schanne, 1986; Williams et al., 1998). In this line, Ho et al. (2020) have shown that haploinsufficiency of C9orf72 impairs LTP in the dentate gyrus (DG-LTP) and LTD in the hippocampal CA1 area (CA1-LTD) as well as adult neurogenesis in the hippocampus.

Mitochondria are organelles found in all cells and they are highly abundant at axonal terminals and dendrites of neurons (Mattson, 2007). Mitochondria regulate calcium homeostasis by removing calcium from the cytoplasm in response to calcium influx into the cell, and they release calcium in response to certain stimuli, such as synaptic transmission (Simpson, 2000; Mattson, 2007). This is accompanied by energy production (ATP) and it also plays a role in redox signaling. This makes mitochondria pivotal in the regulation of synaptic transmission and maintenance of neuronal integrity and function (Mattson et al., 2008; Cheng et al., 2010; Amaral and Pozzo-Miller, 2012; Ivannikov et al., 2013; Manczak et al., 2013; Brot et al., 2014; Su et al., 2014). In dendrites, mitochondria are located mainly in the dendritic shafts and are also found to be associated with spines (Cameron et al., 1991; Popov et al., 2005; Valenti et al., 2014). In response to synaptic stimulation, mitochondria are redistributed toward dendritic protrusions to enhance their activity. Li et al. (2004) reported that decreasing the dendritic mitochondrial content led to a loss of synapses and spines, whereas the number of spines and synapses significantly increased by the accumulation of mitochondria in the dendrites. Furthermore, new spine and synapse formation are enhanced by the aggregation of mitochondria in dendrites. Thus, there are mechanisms for mutual regulation of synaptic plasticity and mitochondrial distribution and activity.

In C9orf72-ALS, mitochondrial dysfunction has been observed in different models and systems. Dafinca et al. (2016) reported that MNs derived from C9orf72 patients show swollen mitochondria and elevated levels of cytochrome c. In addition, a significant reduction in the mitochondrial membrane potential was observed. This indicates a reduced capacity to regulate cytosolic calcium (Dafinca et al., 2016). A few years later, the same group observed that C9orf72 MNs present a low capacity to uptake mitochondrial Ca^2+^ compared with control and corrected MNs. This contributed to glutamate excitotoxicity (Dafinca et al., 2020). Glutamate toxicity is an important feature in neurodegeneration and glutamate regulates synaptic plasticity and modifies cellular energy metabolism (Mattson et al., 1995). Glutamate plays a critical role in synaptic plasticity by activating receptors coupled to calcium influx (Mattson, 2007). In addition, it regulates downstream signaling-pathways *via* activation of kinases (such as PKA, PKC, and ERKs) and transcription factors that are important for long-term alterations of plasticity (Roberson et al., 1999). The protein activation processes are often ATP-dependent, and glutamate also stimulates an increase in mitochondrial oxygen consumption and thereby ATP production (Schuchmann et al., 2005; Mattson, 2007). These features are altered in C9orf72-ALS pathology.

Another study has suggested that mitochondrial ATP production is dysregulated in C9orf72 disease (Mehta et al., 2021). This source of energy is necessary for membrane potential generation, synaptic vesicle recruitment and release, as well as protein phosphorylation reactions (Stefani et al., 1997; Attwell and Laughlin, 2001; Valenti et al., 2014). All of these processes are critical for neuroplasticity and can be modified by changes in ATP production and release (Mattson and Liu, 2003). Mehta et al. (2021) have shown that C9orf72 MNs have shorter axons caused by altered mitochondrial bioenergetic function (Mehta et al., 2021). The authors found reduced gene expression of mitochondria encoded electron transport chain transcripts in MNs-iPSCs from C9orf72 patients. These findings were confirmed in ventral horn spinal MNs of C9orf72-ALS post-mortem tissue. Moreover, the authors demonstrated that the fast axonal transport of mitochondrial cargo was impaired, which is dependent on mitochondrial ATP (Zala et al., 2013). This study showed that axonal phenotypes in C9orf72-ALS were associated with concomitant metabolic dysfunction, owing to defective mitochondrial respiration.

In this line, Choi et al. (2019) established a mouse model of C9orf72, in which poly (GR) was expressed in the brain in a spatially and temporally controlled manner. These authors found that poly (GR) accumulates in the soma and dendrites of neurons in an age-dependent manner. Poly (GR) expression induced ALS/FTD-associated synaptic dysfunction and social behavioral deficits as well as neuronal cell loss, microgliosis, and DNA damage. The increased DNA damage is probably a consequence of compromised mitochondrial morphology and function, which may result in increased oxidative stress that in turn causes DNA damage. This was also observed in C9orf72 iPSC-derived motor neurons (Lopez-Gonzalez et al., 2016). More importantly, poly (GR) preferentially bound to ATP synthase F1 subunit alpha (Atp5a1), which is a subunit of mitochondrial complex V. It has been observed that this protein expression was decreased in poly (GR)-expressing primary cortical neurons cultured from CamKII: (GR) 80 mice, and its expression was reduced in the cortex of 6-months old CamKII: (GR) 80 mice and in the frontal cortex of patients with C9orf72-related ALS/FTD. Other mitochondrial proteins’ expression remained unchanged (Choi et al., 2019). Furthermore, recent *in vitro* studies showed that the reduction of dendritic mitochondrial content through increased mitophagy leads to inhibition of dendrite growth during neuronal polarization (Brot et al., 2014) and to dendrite shortening in mature neuronal cultures (Cherra et al., 2013). Taken together, sufficient dendritic mitochondrial content is required for proper development and maintenance of dendrites, as well as synapse and spine formation. However, the functional role of mitochondria in dendritic protrusions remains to be determined. As in axonal growth cones (Morris and Hollenbeck, 1993), it is possible that changes in ATP demand and required calcium buffering capacity underlie the number of mitochondria in growing spines.

Finally, we want to highlight that dendritic spines rarely contain mitochondria, thus the spine pathology observed in C9orf72 disease may also involve alterations in other organelles near the spines. These include smooth endoplasmic reticulum (SER) or endosomal multivesicular bodies (Spacek and Harris, 1997). Although the contribution of these organelles to neurodegeneration is unclear, they have been studied to a much lesser extent than mitochondria dysfunction and changes in spine distribution. Spine organelles alterations, postsynaptic cell damage, and excitotoxic caused by excessive presynaptic glutamate release are features observed in C9orf72-ALS pathology.

## Involvement of Neuronal Cytoskeleton in C9orf72 Plasticity

Like any other eukaryotic cells, neurons are dependent on their cytoskeleton to maintain their shape, promote cell motility, and to organize their intracellular components (Pollard, 2003). The main components of cytoskeleton include actin, neurofilaments (NFs), and microtubules (MTs). Cytoskeleton dynamics are crucial to promote intracellular transport and to provide structural scaffolding for specialized structures. These include the axonal initial segment, axon and, presynaptic boutons. Dendrites are supported by microtubules, whereas filopodia and simple spines rely on actin remodeling (Neukirchen and Bradke, 2011).

Recent studies have shown that mutations causing neurodegenerative disorders are associated with dysfunction of cytoskeletal components (McMurray, 2000). These mutations often cause the formation of protein aggregates such as tau in AD (Matsuo et al., 1994; Castellani, 2020), alpha-synuclein in PD (Spillantini et al., 1997), Huntingtin (Arrasate and Finkbeiner, 2012), and TDP-43 in ALS (Neumann et al., 2006) and may influence vesicular biogenesis, trafficking and affect synaptic transmission (Table 1). Moreover, these mutations can initiate a cascade of events that include mitochondrial dysfunction, oxidative stress, activate DNA damage response and cause neuronal death. Thus, the dysfunction of the cytoskeleton is likely a common feature observed in several neurodegenerative diseases (McMurray, 2000; Table 1). The dysregulation of cytoskeleton components in C9orf72 pathology is discussed as follows.

**Table 1:** Common disease mechanisms of neurodegenerative diseases will lead to synaptopathy.

Disease	Protein misfolding	Neuronal loss	Mitochondria dysfunction	Glutamate excitotoxicity	Proteostasis defects	Electrophysiology defects	Synaptic impairment
Alzheimer’s disease	Tau (Matsuo et al., 1994; Castellani, 2020)	Yes (Terry et al., 1991; Kril et al., 2002; Selkoe, 2002; Coleman et al., 2004)	Yes (Swerdlow, 2018; Wang et al., 2020b)	Yes (Hynd et al., 2004; Binvignat and Olloquequi, 2020)	Yes (Oddo, 2008; Gong et al., 2016)	Yes (Fernandez-Perez et al., 2020; George et al., 2020)	Yes (DeKosky and Scheff, 1990; Games et al., 1995; Mucke et al., 2000; Scheff and Price, 2003; Coleman et al., 2004; Bjorklund et al., 2012)
Parkinson’s disease	Alpha synuclein Lewy body (Spillantini et al., 1997)	Yes (Janezic et al., 2013)	Yes (Reinhardt et al., 2013; Pickrell and Youle, 2015)	Yes (Binvignat and Olloquequi, 2020; Wang et al., 2020a)	Yes (Liu et al., 2012)	Yes (Creed et al., 2020; Tubert and Murer, 2020)	Yes (Janezic et al., 2013; Rockenstein et al., 2014)
Huntington’s disease	Huntingtin (Arrasate and Finkbeiner, 2012)	Yes (Guidetti et al., 2001; Klapstein et al., 2001)	Yes (Guidetti et al., 2001; Klapstein et al., 2001)	Yes (Binvignat and Olloquequi, 2020)	Yes (Lynch-Day et al., 2012)	Yes (Klapstein et al., 2001; Ribchester et al., 2004)	Yes (Graveland et al., 1985; Ferrante et al., 1991; Guidetti et al., 2001; Klapstein et al., 2001)
Amyotrophic Lateral Sclerosis	RAN translation DPR (Ash et al., 2013; Mori et al., 2013), TDP-43 (Neumann et al., 2006), FUS (Vance et al., 2009), SOD1 (Bosco et al., 2010)	Yes (Brettschneider et al., 2014; Saberi et al., 2015)	Yes (Choi et al., 2019; Dafinca et al., 2020)	Yes (King et al., 2016; Binvignat and Olloquequi, 2020)	Yes (Kabashi and Durham, 2006; Maurel et al., 2018)	Yes (Selvaraj et al., 2018; Dyer et al., 2020)	Yes (Jiang et al., 2016; Lopez-Erauskin et al., 2018; Choi et al., 2019; Just-Borras et al., 2019; Petel Legare et al., 2019; Dyer et al., 2020; Jensen et al., 2020)

The actin dynamics in neurons regulate extension and direction of axon growth, whereas in the dendritic spines, it plays a fundamental role in assembly and disassembly of synapses (Coles and Bradke, 2015).

Dysregulation of actin remodeling is a process thought to be implicated in ALS neurodegeneration (Hensel and Claus, 2018). In C9orf72 pathology, actin remodeling is also impaired. Giampetruzzi et al. (2019) have shown that actin homeostasis is disrupted in C9orf72-ALS. In this study, the authors transfected primary motor neurons with a synthetic construct expressing 80× GGGGCC repeats (G4C2-80). No differences in F-actin levels at the growth cone were found between conditions, however, the authors observed promoting actin filament assembly in fibroblasts from ALS patients can alleviate defects in nuclear-cytoplasmic transport defects (Giampetruzzi et al., 2019).

In order to understand the key regulators of actin dynamics in C9orf72-ALS, Sivadasan et al. (2016) over-expressed influenza hemagglutinin (HA)-tagged human C9orf72 protein and immunoprecipitated interacting proteins from mouse neuroblastoma NSC-34 cells. These authors found that cofilin, Arp2/3, and coronin were among statistically significant interaction partners of C9orf72. Special attention needs to be given to cofilin which promotes actin assembly or disassembly depending on the concentration of the former. This concentration is relative to actin, but also to other actin-binding proteins (Bravo-Cordero et al., 2013). The activity of cofilin is dependent on its phosphorylation at Ser3 residue, which inactivates its function in F-actin assembly (Moriyama et al., 1996). The authors observed that the overexpression of C9orf72-HA protein reduced phospho-cofilin (Ser3), while knockdown increased cofilin phosphorylation at Ser3 without changing total cofilin levels. These results were replicated in human cells with *C9orf72* intronic expansion and in post-mortem cerebellar brain tissue from C9orf72-ALS patients (Sivadasan et al., 2016). Finally, the study shed some light on the influence of C9orf72 on actin dynamics. In this regard, Sivadasan et al. (2016) found that the number of newly generated actin filaments was reduced in C9orf72-depleted motor neurons which was accompanied by a significant reduction in the velocity of actin movement in axonal growth cones in motor neurons.

In the same line, Radwan et al. (2020) have demonstrated that Arginine-rich DPRs (PR and GR) impede the assembly of the actin cytoskeleton and this is correlated to a significant reduction in F-actin levels. Thus, modulation of the actin dynamics could represent potential therapeutic strategies for C9orf72-ALS pathology.

NFs are also known to contribute to the growth and stability of axons in both central and peripheral nerves, as well as to maintain mitochondrial stability (Gentil et al., 2015) and MT content (Bocquet et al., 2009). Additionally, NFs are also integral components of synapses. This is based on Cochard and Paulin (1984) study on mouse embryonic neuronal axons where NFs were present, pointing out their role in early development. Other studies have also shown that synapses contain a unique pool of NFs (Yuan et al., 2015a, b). These NFs isolated from brain synaptosomes were distinguishable both morphologically and biochemically from other NFs from different parts of the neuron (Yuan et al., 2003).

More interestingly, it has been shown that changes in phosphorylation of synaptic neurofilament light chain (NfL) are associated with calcium/calmodulin-dependent protein kinase II activation during modulation of LTP (Hashimoto et al., 2000), which it is altered in C9orf72-ALS (Ho et al., 2020). These results suggest that disturbed LTP may be caused by alterations in synaptic neurofilament proteins in C9orf72-ALS. In addition, it is important to highlight the role of NFs isoforms in regulating glutamatergic and dopaminergic synaptic neurotransmission (Schwartz et al., 1995). In this respect, several studies have demonstrated that NfL are expected to stabilize NMDA receptors within the neuronal plasma membrane (Ehlers et al., 1998; Ratnam and Teichberg, 2005; Gafson et al., 2020). These results are supported by observations that the cellular distribution of the NMDA GluN1 receptors could be linked to the phosphorylation states of NFs subunits (Ehlers et al., 1998; Terry-Lorenzo et al., 2000).

Taken together, any alteration in NFs subunits could be disrupting the synaptic scaffolding, leading to changes in LTP and LTD, and neurotransmission. Ultimately, these changes can lead to neuronal and cognitive changes as observed in C9orf72-ALS neurodegeneration. In this line, NFs have been used as a sensitive biomarker for neurodegeneration in different neurological disorders, including in C9orf72-ALS (Gendron et al., 2017; Khalil et al., 2018). During axonal degeneration, NFs are released into cerebrospinal fluid (CSF) and blood (Zucchi et al., 2020). In C9orf72 patients, phosphorylated neurofilament heavy (pNfH) proteins are found in the CSF and predict disease progression and survival. Patients expressing higher levels of pNFH exhibit faster disease progression and shorter survival (Gendron et al., 2017). Benatar et al. (2019) also investigated the phosphorylation status of NfH, as well as NfL in C9orf72 patients (Benatar et al., 2019). The authors observed an increase in the absolute levels of NfL proteins, however, pNfH levels remained unchanged in serum. No differences in both neurofilaments were found in CSF or blood. Specifically, in C9orf72 patients, NfL and pNfH were elevated ~3.5 years prior to phenoconversion, highlighting the importance of tracking neurofilament proteins as biomarkers of C9orf72-ALS (Benatar et al., 2019). In addition, Lu et al. (2015) have shown an increase of the NfL isoform and a modest upregulation of heavy and medium NF chain subunits in the blood of ALS patients. Thus, the levels of NFs could help to understand the relationship between genotype, the duration of the pre-symptomatic phase and predict survival of C9orf72-ALS.

At last, during axonal degeneration, NF accumulates in bundles and forms spheroid structures known as axonal swellings or blebs. These abnormal structures recruit hyperphosphorylated NfH and NfM in axons and gradually disrupt axonal transport, causing axonal fragmentation (Zucchi et al., 2020).

## Axonal Transport Degeneration in C9orf72

Axonal transport is a key cellular process by which RNA, organelles, synaptic vesicles, proteins and, lipids are trafficked in bidirectional ends (Sleigh et al., 2019). Both anterograde and retrograde transports are MT-dependent and key for MN survival, maintenance, and functionality (Chevalier-Larsen and Holzbaur, 2006). The length of the MNs axons is highly dependent on cytoskeletal architecture and axonal transport stability. In C9orf72-ALS pathology the cytoskeleton integrity is compromised, leading to a disruption in the axonal transport necessary to maintain synapse integrity. Indeed, distal axonopathy has been observed in many neurodegenerative diseases, including ALS (Sleigh et al., 2019). This leads to defective axonal transport, a key initiating contributor to the selective vulnerability of motor nerves (Baldwin et al., 2016). The expression of the different pathogenic ALS mutations in *SOD1* (Zhang et al., 1997; Williamson and Cleveland, 1999), *TARDBP* (Wang et al., 2013; Magrane et al., 2014), *FUS*, and *C9orf72* (Baldwin et al., 2016) contributes to axonal transport defects, which are early pathology events that precedes neuronal loss and clinical symptoms.

As we have previously described, actin dynamics and neurofilaments alterations have been observed in C9orf72-ALS pathology. These observations could explain the disruption of cytoskeleton integrity and/or MT-dependent transport mechanisms which lead to the inability of MNs to supply their synapses with essential components and/or to convey information back to the cell body, potentially triggering the degeneration processes associated with C9orf72-ALS.

In this line, it has been recently proposed that mutations in *NFL* and *NFH* genes might affect the axonal integrity of MNs by altering the transport of other essential components in ALS (Zucchi et al., 2020). Two experiments were key to demonstrate their contribution to the disease. Lee et al. (1994) overexpressed NfL in mice and they observed degeneration of MNs, perikaryal and axonal swellings with the presence of intermediate filament spheroids, and NMJ denervation presenting as muscle atrophy. In the same hypothesis line, Julien et al. (1995) overexpressed NfH in mice observing swellings of proximal axons in the spinal cord, progressive axonopathy, and atrophy of muscle fibers. More interestingly, in this study not only the axonal transport of NF proteins was altered, but also actin, tubulin, and mitochondria.

In addition, other proteins have been involved in the axonal transport defects in C9orf72 models. Liang et al. (2019) observed that transgenic *C9orf72* BAC mice presented a loss of function of C9orf72 protein as well as Smcr8. Both proteins form a complex (C9orf72-Smcr8) which associates with dynein causing the disruption in the axonal transport in MNs and impairment of retrograde transport resulted in stalling of autophagosomes. Furthermore, abnormal axon swellings formation was found in the spinal cord and NMJ in these mutant mice.

Moreover, in the 2016’s study, Baldwin and colleagues observed that *Drosophila* G4C2 overexpressing lines presented defects in axonal transport which increased the stationary mitochondria (Baldwin et al., 2016). They observed that the presence of DPRs, especially the PR-36, was causing a severe disruption of vesicle transport leading to mitochondria stalling. All these results together question whether mutations in NFs lead to a secondary effect or contribute to worsening the disease phenotype in synergy with C9orf72 haploinsufficiency and/or DPRs accumulation.

Recently, Fumagalli et al. (2021) demonstrated the existence of inhibitory interactions of arginine-rich DPRs with axonal transport machinery in C9orf72-associated ALS/FTD. In this study, the authors observed impaired microtubule-based transport in iPSC-derived MNs from C9orf72-ALS/FTD patients, including elevated stalled cargoes. This is in line with Abo-Rady et al. (2020) who reported that lysosome trafficking is also impaired in neurons with the C9orf72 repeat expansion. Moreover, Fumagalli et al. (2021) also showed that when control iPSC-derived motor neurons and *Drosophila* neurons were exposed to poly-PR and poly-GR, comparable effects in microtubule transport deficits were found. Protein interaction studies performed in MNs and post-mortem patient tissues revealed an association of arginine-rich DPRs with kinesin-1, dynein, and, microtubules. More importantly, the single-molecule imaging of purified components demonstrated that the arginine-rich DPRs directly impede motility by binding both the unstructured tubulin tails of microtubules and dynein and kinesin-1 motor complexes. However, while the DPRs increase the binding to microtubules and the duration of its processive runs to the active form of human kinesin-1 isoform (KIF5B), DPRs showed opposite results with dynein in a dose-dependent manner. These results support a direct inhibitory effect of arginine-rich DPRs on axonal transport and possibly different processes may contribute to trafficking defects observed in C9of72-ALS/FTD neurons. Nonetheless, these data highlight the relevance of targeting the cytoskeleton integrity as a therapy for C9orf72-ALS pathology.

## C9orf72 Common Disease Mechanisms

### Repeat Expansion Diseases

**Figure 2:**
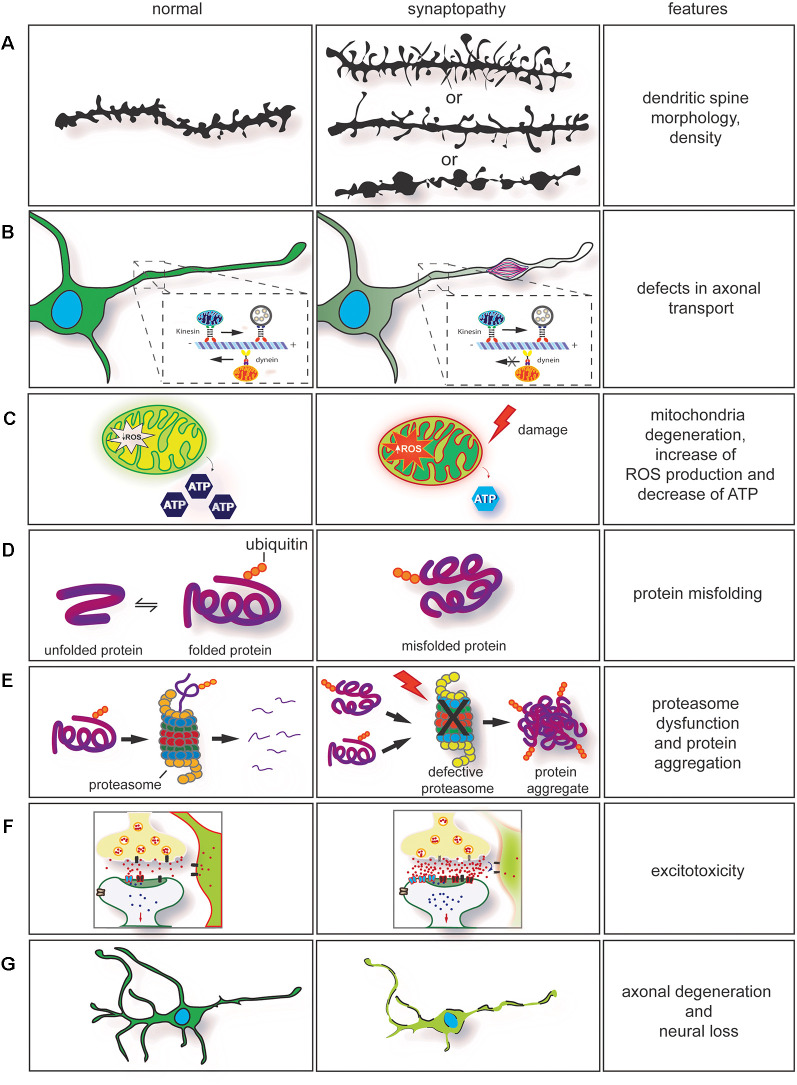
Dysregulation of key cellular mechanisms leads to synaptopathy. **(A)** Drawing of dendritic spines in controls (left) and in neurological diseases (right). The morphology and density of dendritic spines are altered in several neurodegenerative conditions. In synaptopathies (**A**, middle section), immature spines are normally long and thin, and density can be increased or decreased. On few occasions, neurite swellings with long and thin spines are observed. **(B)** Axonal transport impairment in neurodegenerative conditions leads to synaptopathy. Retrograde and anterograde transport disruption leads to stalling of mitochondria transport. Furthermore, rearrangement of neurofilaments (NFs) and damage caused by the neurodegeneration process will lead to formation of axonal swelling impairing axonal transport. **(C)** Mitochondria dysfunction [ATP production, change in homeostasis, and increase of reactive oxygen species (ROS)] are observed in neurodegenerative conditions. These contribute to synaptic dysfunction. **(D)** Mutations in genes involved in neurodegenerative conditions often cause protein misfolding and accumulation in neurons. **(E)** In association with proteasome dysfunction, protein misfolding will form insoluble protein accumulation, enhancing the neurodegeneration process. This contributes to axonal degeneration and axonal transport impairment. **(F)** Impairment in axonal transport, alteration in calcium buffering, mitochondria degeneration, formation of ROS associated with dysregulation of synaptic proteins can enhance glutamate excitotoxicity. **(G)** Neuronal degeneration and neuronal loss are also observed in neurodegenerative conditions. They are a result of the neurodegeneration process.

C9orf72 mechanism of disease is unique within the ALS spectrum as the three plausible disease mechanisms have been observed in different systems including cellular and animal models. Furthermore, downregulation of C9orf72 protein, the presence of RNA foci and DPRs have been observed in patient’s post-mortem samples and iPSCs derived neurons (Balendra and Isaacs, 2018; Shi et al., 2018; Westergard et al., 2019; Dafinca et al., 2020). Despite the unusual pathomechanism of disease, C9orf72 holds similarities with other expansion diseases (Rodriguez and Todd, 2019), such as Myotonic Dystrophy type 1 (DM1), Myotonic Dystrophy type 2 (DM2; Figure 2), and Friedreich ataxia (FA; Nguyen et al., 2019; Table 2).

**Table 2:** Common molecular features observed in expansion diseases.

Disease	Gene	Expansion	Repeat location	RAN translation-toxicity	RAN translation	RNA toxicity	Loss of function
*Friedreich’s ataxia*	FRDA	GAA/TTC 55–200	Intron	No	N/A	No	Downregulation of frataxin
*Myotonic Dystrophy type 1*	DMPK	CTG/CAG 50–1000	3’UTR	No	Sense [poly(C), poly(A), poly(L)] Antisense [poly(Q), poly(A), poly(S)]	Yes Recruitment of MBNL1 into RNA foci	Downregulation of MBNL1 and DMPK
*Myotonic Dystrophy type 2*	CNBP/ZNF9	CCTG/CAGG 55–11000	Intron 1	Yes	Sense [poly(LPAC)] Antisense [poly(QAGR)]	Yes Disbalance of MBNL1 and CUGBP1 and recruitment of MBNL1 into RNA foci	Downregulation of CNBP
*C9orf72*	C9orf72	GGGGCC/ GGCCCC 100–4500	Intron 1	Yes	Sense [poly (GP), poly(GA), poly (GR)] Antisense [poly (GP), poly (PA), poly (PR)]	Yes Recruitment of RNA binding proteins (hnRNP-H) into RNA foci	Downregulation of C9orf72

DM1 is a genetic disease caused by myotonia, cardiopathy, muscular dystrophy, and cataracts (Meola and Cardani, 2015). It is caused by the expansion of CTG in the 3’ untranslated region (UTR) of myotonic dystrophy protein kinase (DMPK; Brook et al., 1992). RNA toxicity is the main pathological feature observed in DM1, which causes the formation of RNA foci and recruitment of an RNA-binding protein known as muscle blind-like 1 (MBNL1) protein, a protein involved in alternative splicing (Miller et al., 2000). The sequestration of MBNL in RNA foci leads to upregulation of CUG-binding protein (CUGBP1; Lin et al., 2006). This imbalance of MBNL and CUGBP1 causes splicing defects on several mRNAs (Philips et al., 1998). DM1 seems to produce RAN translation products, including poly (C), poly (A), and poly (L) from the sense strand, and poly (Q), poly (A), and poly (S) from the antisense strand. However, it is unclear whether these DPR products are pathogenic (Zu et al., 2017). Downregulation of DMPK leads to mild myopathy in knockout mice, suggesting it plays a minor role in the disease (Jansen et al., 1996).

DM2 is less frequent than DM1 and the clinical presentation varies (Meola and Cardani, 2015). DM2 is caused by an expansion of CCTG repeats in the intron of cellular nucleic acid binding protein (CNBP), also known as Zinc finger factor 9 (ZNF9). The disease mechanism of DM2 is similar to DM1 and is caused by RNA toxicity with the formation of RNA foci with recruitment of MBNL1 protein and disbalance between MBNL1 and CUGBP1 (Jones et al., 2011). Furthermore, RAN translation produces the peptides poly (LPAC; sense) and poly (QAGR; antisense). Contrary to DM1, DM2 RAN translated peptides are present in autopsy tissues from patients with DM2 and seem to be toxic in cells independent of RNA gain of function (Zu et al., 2017). Haploinsufficiency of CNBP causes abnormal feature reflecting DM2 phenotype in murine models (Chen et al., 2007).

Friedreich’s ataxia (FRDA) is another repeat expansion disease caused by GAA repeats in intron 1 of *frataxin (FXN)* gene, which encodes for a mitochondrial protein. FRDA affects the peripheral and central nervous system (Lin et al., 2017). The expansion causes haploinsufficiency of *FXN* leading to epigenetic modifications on the gene, reducing transcription of frataxin protein. In addition to common disease mechanisms, DM and FDRA present synaptic dysfunction (Hernandez-Hernandez et al., 2013; Lin et al., 2017). Transgenic models for myotonic DM1 revealed that the CTG expansion leads to upregulation of RAB3A protein, leading to dysfunction in neurotransmission and mouse behavior (Hernandez-Hernandez et al., 2013). In contrast, FA’s transgenic mice presented early VGLUT1 depletion and dysregulated synaptic input deficits and cerebellar circuit (Lin et al., 2017).

### Neurodegenerative Diseases

As we have previously described, C9orf72 localization and function and DPR localization at synapses indicated C9orf72 disease is a synaptopathy within the neuromotor system. Moreover, other neurological diseases have also shown features of synaptopathy with altered synaptic structure and function. Among these neurodegenerative conditions, we include Parkinson’s disease (PD), Huntington’s (HD), prion pathologies, and Alzheimer’s disease (AD; Table 1). Early onset neurological diseases such as schizophrenia (SCZ), bipolar disorder (BP), and autism spectrum disorders (ASD) are also synaptopathies (Lepeta et al., 2016).

By comparing these neurodegenerative diseases’ pathology and progression, common features among synaptopathies diseases are observed (Table 1). This allows us to deepen our understanding of the neurodegeneration process and may help us to design therapies that target one pathophysiological pathway common to other synaptopathies.

In AD, a progressive loss of dendritic spines in hippocampal pyramidal neurons (Scheibel, 1979b; Ferrer and Gullotta, 1990), dentate granule cells (de Ruiter and Uylings, 1987; Gertz et al., 1987; Einstein et al., 1994), and neocortical pyramidal neurons (Scheibel, 1979a; Catala et al., 1988; Baloyannis et al., 1992) have been observed. Moreover, synapse loss in AD is accompanied by a compensatory increase in synaptic bouton’s size (Scheff et al., 1990; Scheff and Price, 1993). A similar neuronal loss and extensive dendritic spine loss in the cortex have also been found in FTD (Baloyannis et al., 2001), in neocortical neurons in Pick’s disease (Ferrer, 1999), and ALS (Ferrer et al., 1991). Moreover, a reduction in dendritic spines in dopaminergic projections of striatal neurons in PD (McNeill et al., 1988) and in *locus ceruleus* and substantia nigra in rodent models for PD (Shors et al., 2001) have been also observed. Furthermore, a decrease in spine density has been found in severe HD cases (Ferrante et al., 1991). This observation was confirmed in the striatal and cortical regions of HD transgenic mice (Guidetti et al., 2001). However, an increased spine density has also been observed in HD models (Ferrante et al., 1991).

Finally, we also want to highlight that neuronal loss, mitochondria dysfunction, glutamate excitotoxicity, proteostasis defects, and synaptic impairment are other features common to other synaptopathies (Table 1). Whether these events are the endpoint of signaling cascades or common mechanisms of neurodegenerative diseases, it remains to be elucidated.

## Conclusion

In this review article, we addressed how the expansion in the C9orf72 gene leads to the formation of RNA foci, DPRs, and downregulation of C9orf72 transcripts. Furthermore, we discuss how C9orf72 may have a function at synapses and how the convergence of a wide range of cellular defects is observed in other neurodegenerative conditions. Dysregulation of key cellular mechanisms, such as dendritic spine morphology defects, defects in the axonal transport and axonal degeneration, mitochondria degeneration, protein misfolding, protein aggregation and proteasome dysfunction, excitotoxicity, and neuronal loss have always been addressed separately. However, these disease features commonalities are often presented together, and this evidence can no longer be overlooked. These converging mechanisms can provide insights into the pathogenic pathways leading to synaptopathy and will aid better identification of disease hallmarks of neurodegenerative diseases. Furthermore, it may offer potential targets for intervention to prevent synaptic degeneration in ALS caused by C9orf72 repeat expansion.

## Author Contributions

AN: writing—original draft preparation, reviewing, and editing. NA: wrote the first draft of the manuscript, supervision, conceptualization, and reviewing. All authors contributed to the article and approved the submitted version.

## Conflict of Interest

The authors declare that the research was conducted in the absence of any commercial or financial relationships that could be construed as a potential conflict of interest.
